# Supplementary material for: A contact-electro-catalysis process for producing reactive oxygen species by ball milling of triboelectric materials
Source: Nat Commun. 2024 Jan 26;15:757. doi: 10.1038/s41467-024-45041-4 (PMC10810876; doi:10.1038/s41467-024-45041-4)
Supplement: Supplementary file 1 — Supplementary Information [file 41467_2024_45041_MOESM1_ESM.pdf]

Supplementary Information

A contact-electro-catalysis process for producing reactive oxygen species by ball milling of triboelectric materials

Authors: Ziming Wang[1,2,†], Xuanli Dong[1,2,†], Xiao-Fen Li[3,†], Yawei Feng[1,4], Shunning Li[5], Wei Tang[1,2,*], Zhong Lin Wang[1,2,6,*]

Affiliations:

[1] CAS Center for Excellence in Nanoscience, Beijing Institute of Nanoenergy and Nanosystems, Chinese Academy of Sciences; Beijing, 100140, China.

[2] School of Nanoscience and Engineering, University of Chinese Academy of Sciences; Beijing, 100049, China.

[3] Key Laboratory of Advanced Materials (MOE), School of Materials Science and Engineering, Tsinghua University; Beijing 100084, China.

[4] Department of Mechanical Engineering, City University of Hong Kong, Hong Kong 999077, P. R. China

[5] School of Advanced Materials, Shenzhen Graduate School, Peking University, Shenzhen, 518055, China.

[6] School of Materials Science and Engineering, Georgia Institute of Technology, Atlanta, GA 30332-0245, USA.

† These authors contributed equally to this work.

*Corresponding author. Email: tangwei@binn.cas.cn (W.T.); zhong.wang@mse.gatech.edu (Z.L.W.)

The PDF file includes:

Supplementary Note 1. Analysis on contact-electrification pairs during liquid-assisted grinding process

Supplementary Note 2. Theoretical analysis on ball motion and impact during a planetary milling process

Supplementary Note 3. Effect of ball sizes on CEC efficiency

Supplementary Note 4. Details of DFT simulations

Supplementary Table 1. Calculated specific value of impact energy of 5-mm PTFE balls under various revolution speeds

Supplementary Fig. 1 | Schematic illustration of measuring transferred charges of a pristine polymer that is repeatedly contacted with water by a single-electrode mode triboelectric nanogenerator (SE-TENG)

Supplementary Fig. 2 | Measured transferred charges of a pristine polymer-based SE-TENG that is repeatedly immersed in ultrapure water

Supplementary Fig. 3 | Schematic illustration and corresponding measured profiles of the effect of introducing ter-butanol on the production of DMPO adducts

Supplementary Fig. 4 | Thermal stability test of methyl orange aqueous solution

Supplementary Fig. 5| The chemical structure of degradation products during the CEC degradation of methyl orange aqueous solution as identified by mass-spectroscopy
Supplementary Fig. 6 | Optical and corresponding Image J-processed images of PTFE milling balls before and after reactions
Supplementary Fig. 7 | Characterization of PTFE milling balls before and after contact-electro-catalysis
Supplementary Fig. 8 | Recyclability of PTFE milling balls during contact-electro-catalysis
Supplementary Fig. 9 | Investigations on degradation by ZrO_2 and its CE ability
Supplementary Fig. 10 | Grinding at 50 RPM with prolonged milling time for methyl orange aqueous solution degradation
Supplementary Fig. 11 | Magnified illustration of degrading methyl orange aqueous solution in PP group under different revolution speeds
Supplementary Fig. 12 | Theoretical analysis of electron transfer process between PTFE and O_2
Supplementary Fig. 13 | Schematic illustration for the theoretical analysis of a planetary milling process
Supplementary Fig. 14 | Numerically calculated ball motion and impact under various revolution speeds
Supplementary Fig. 15 | Calculated evolution of impact energy and frequency when the diameter of PTFE balls varies from 4 to 8 mm

Supplementary Note 1:

In a typical liquid-assisted grinding (LAG) setup, frequent contact-separation cycles could take place between milling balls and vials, grinding media (liquids) and balls/vials, as well as among balls. All these collisions are capable of inducing contact-electrification (CE) effects as long as contacting pairs exhibit different electron-withdrawing (EW) abilities, using different materials for example. However, abrasions are inevitable if milling vials and balls are made of different materials, which is supposed to induce breakages of chemical bonds, and thus complicating the interpretation of catalytic mechanism. In order to unambiguously reveal the contribution of CE effect, the same material was employed to fabricate milling vials and balls in each group as a mean to minimize the abrasion and it-derived influences. Besides, owing to the fact that CE can hardly happen between same materials, the utilization of same material in each group actually simplifies the analysis on contribution from CE as CE between vials and balls as well as among balls are negligible. The only CE pairs in LAG are liquids/vials and liquids/balls, which enables quantitative comparison of contact-electro-catalysis efficiency among materials with various CE abilities. Hence, the milling vials and balls were made of the same material in each group.

Supplementary Note 2:

In accordance with previous studies, several reasonable assumptions were introduced before we establish a qualitative model to reveal the variation tendency of ball motions under different milling parameters.[1] First of all, an entire ball milling process that involves a number of balls was regarded as a linear cumulative result of single ball motions. Further, the single ball was treated as a mass point that no slip would happen when it moves along with the vial wall. At last, the motion of ball was assumed to be periodic which includes three stages: Initiation, detachment, and re-initiation. The detail investigations are as follow:

As depicted in Supplementary Fig. 13, a Cartesian coordinates system was established here with its origin (O) located at the center of the planetary disk. The distance between the origin and the center of vials (C) is labeled by R_d, and the radius of vials is R_v. The disk is supposed to rotate anticlockwise with a rotation speed of ω_d, while the revolution speed of vials is defined as ω_v in a reverse direction. At the initial stage (t=0), coordinates of the center of vials and ball are defined as C_0 (X_{c0}, Y_{c0}) and B_0 (X_0,Y_0), respectively. Before the milling ball detach from the wall of vials ($0<t<T_1$), the position vector of ball at any time instant t_i could be described as:

$$x_1 = r_d \cos\omega_d t_i + r_v \cos(\omega_d - \omega_v)t_i \quad (1)$$

$$y_1 = r_d \sin\omega_d t_i + r_v \sin(\omega_d - \omega_v)t_i \quad (2)$$

Therefore, the velocity component in the x and y directions can be calculated as follows:

$$v_x = \frac{\mathrm{d}x_1}{\mathrm{d}t} = -\omega_d r_d \sin\omega_d t_i - (\omega_d - \omega_v) r_v \sin(\omega_d - \omega_v)t_i \quad (3)$$

$$v_y = \frac{\mathrm{dy}_1}{\mathrm{d}t} = -\omega_d r_d \cos\omega_d t_i + (\omega_d - \omega_v) r_v \cos(\omega_d - \omega_v)t_i \quad (4)$$

During such period, the force exerted on the ball includes its own gravity and centrifugal force that originates from the rotation of the vial as well as ball. The gravity can be reasonably omitted since it is usually much smaller than the centrifugal force. Thus, the net force applied on the ball can be derived as follows:

$$F = m[r_v(\omega_d - \omega_v)^2 + r_d \omega_d{}^2 \cos\phi] \quad (5)$$

The ball will detach from the vial once $F \leqslant 0$, which enables us to calculate the detaching angle Φ_1:

$$\cos\phi_1 = -\frac{r_v(\omega_d - \omega_v)^2}{r_d \omega_d{}^2} \quad (6)$$

Thus, we can obtain the time span of the initial stage (T_1) by the following:

$$T_1 = \frac{\phi_1}{\omega_v} \quad (7)$$

The detaching speed of ball at this moment can be expressed as:

$$v = \sqrt{\omega_d{}^2 r_d{}^2 + (\omega_d - \omega_v)^2 r_v^2 + 2\omega_d(\omega_d - \omega_v) r_v r_d \cos\phi_1} \quad (8)$$

The component of velocity along axis and the coordinates of ball at this instant can be derived by substituting Equation 7 into Equation 1 to 4, respectively.

So far, aiming for the motion of balls, we can obtain the value of coordinates (x_1, y_1), velocity and its component (v, v_{x1}, v_{y1}) at the end of initial stage by above derivations. The following investigations will focus on the detaching period ($T_1<t<T_1+T_2$).

The following relationship will be satisfied once the ball has attached to the vial wall:

$$(X_2 - X_{c2})^2 + (Y_2 - Y_{c2})^2 = (X_1 - X_{c1})^2 + (Y_1 - Y_{c1})^2 \quad (9)$$

According to previous assumptions, the ball will move straightly once it has detached from the vial. As a consequence, we have:

$$X_2 = X_1 + v_{x1}t_2 \tag{10}$$

$$Y_2 = Y_1 + v_{y1}t_2 \tag{11}$$

Based on Equations 10 and 11, the Equation 9 can be reformulated as:

$$\begin{aligned} &v^2T_2^{\ 2} + 2(x_1v_{x1} + y_1v_{y1})T_2 \\ &-2[x_1r_d\cos(\phi_1 + \omega_dT_2) + y_1r_d\sin(\phi_1 + \omega_dT_2)] \\ &-2T_2[v_{x1}r_d\cos(\phi_1 + \omega_dT_2) + v_{y1}r_d\sin(\phi_1 + \omega_dT_2)] + 2(x_1x_{c1} + y_1y_{c1}) = 0 \end{aligned} \tag{12}$$

The value of X_{c1} and Y_{c1} can be derived based on the geometric relationships:

$$X_{c1} = r_d\cos\theta \tag{13}$$

$$Y_{c1} = r_d\sin\theta \tag{14}$$

$$\theta = \phi_1\frac{\omega_d}{\omega_v} \tag{15}$$

Thus, T_2 is the only unknown parameter in Equation 12, which can be numerically solved. The last stage of ball motion concerning about re-initiation, i.e., the ball will move with vial to its original point to initiate next motion cycle. The time span T_3 of this stage can be derived as:

$$T_3 = \frac{360 - (\phi_1 + \omega_vT_2)}{\omega_v} \tag{16}$$

The period for a complete motion cycle T is expressed as:

$$T = T_1 + T_2 + T_3 \tag{17}$$

And the frequency of ball motion f is:

$$f = \frac{1}{T} \tag{18}$$

The kinetic energy of this ball (E_{k}) can be described as:

$$E_k = \frac{1}{2}mv^2 \tag{19}$$

where m denotes the mass of milling balls. The total power that transferred by the ball can be calculated by:

$$P = fE_k \tag{20}$$

For a typical *Pulverisette 5 Pro* planetary ball mill (*Fritsch* Inc.) with 500 mL milling vials, the frequency of ball motion under various ω_{d} has been calculated and summarized in Supplementary Fig.14a. Taking the 5 mm PTFE milling balls as example, the kinetic energy and total transferred power under various rotation speed have been depicted in Supplementary Fig.14b, and c.

Supplementary Note 3:

Owing to the total weight of milling balls is given as 100 g in this study, the size of milling balls would simultaneously affect the weight of individual ball and the total number of balls. Thus, the impact energy (depend on the weight of individual ball) would increase, while the impact frequency (relevant to the number of balls) would decrease along with the increase of ball sizes. Based on the qualitative model for variation tendency of ball motions in Supplementary Note 2, calculated evolutions of impact energy and frequency when the size of PTFE balls increases from 4 to 8 mm were obtained and exhibited in Supplementary Fig. 15. The revolution speed in calculation is fixed at 350 RPM.

Although an apparently higher collision frequency can be obtained by employing smaller PTFE balls, the impact energy brought by these balls might be insufficient to drive interfacial electron transfer for catalysis. Thus, we expect the impact energy is the limiting factor for degradation at this stage, and the degradation rate would first rise with the increase of ball's size. This is also in accordance with the experimental observations that the degradation rate in 5-mm group is higher than in 4-mm group. However, further increases in ball sizes would bring about a significant decrease in the impact frequency, which is also a key factor that affects the CE-driven electron transfer process. On this condition, the impact energy should already beyond the requirement for driving electron transfer during CE, and the impact frequency is supposed to appear as the limiting factor for degradation. Thus, the degradation rate is expected to decrease with further increase of ball sizes, which is responsible for the result that the degradation rate in 8-mm group is lower than that in 5-mm group.

To wrap up, the evolution of degradation rate could be ascribed to that the impact energy and frequency respectively appears as the limiting factor in different conditions, and a trade-off relationship exists between these two factors. Thus, there exists an optimum ball size for achieving the highest CEC efficiency by balancing collision frequencies and impact energies.

Supplementary Note 4:

Density functional theory (DFT) based on the Perdewe-Burke-Ernzerhof (PBE) generalized gradient approximation[2] with ion-electron interactions described by the projected augmented wave (PAW) method[3,4] as implemented in the Vienna ab initio simulation package (VASP) was employed in the present work. The kinetic energy cutoff is set to 520 eV, A conjugate gradient method was applied for geometry optimization, with a Gaussian smearing width of 0.05 eV.[5,6] The total energy criterion in the electronic self-consistency loop and the force criteria in the ionic relaxation loop was set to 10^{-5} eV and 0.02 eV $Å^{-1}$, respectively. To complement the deficiencies of DFT in treating dispersion interactions, the underlying structural optimizations were carried out including the third-generation (D3) semiempirical van der Waals corrections proposed by Grimme. When calculating the charge density difference, supercells with about 40 atoms were used, in combination with a Γ-centered 2×2×10 k-mesh.

Supplementary Table 1. Calculated specific value of impact energy of 5-mm PTFE balls under various revolution speeds.

Revolution Speeds (RPM)	Impact energy (J)
50	0.08805
100	0.3522
150	0.79245
250	2.20126
350	4.31446

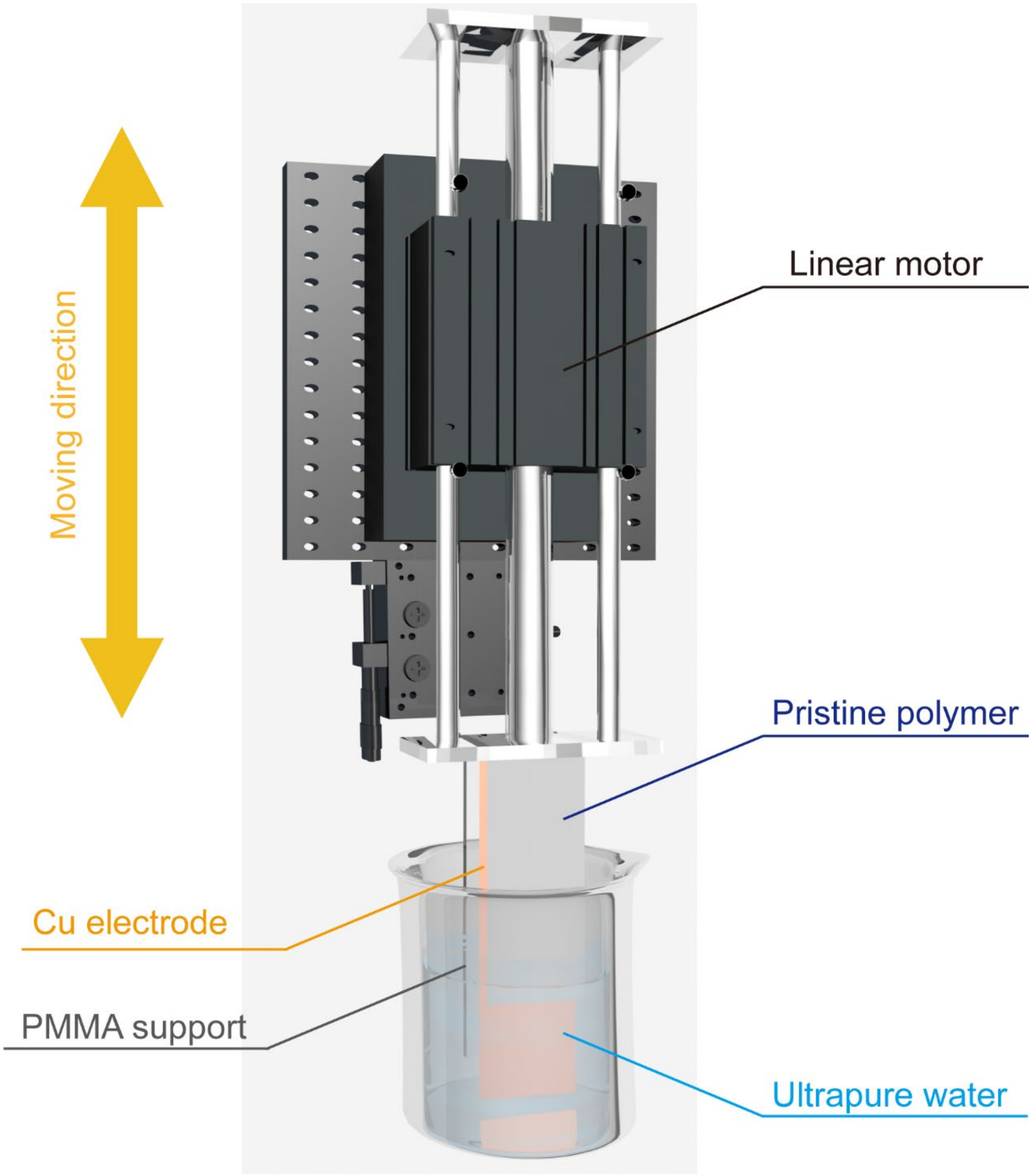

Supplementary Fig. 1 | Schematic illustration of measuring transferred charges of a pristine polymer that is repeatedly contacted with water by a single-electrode mode triboelectric nanogenerator (SE-TENG). Created with SolidWorks and Adobe Illustrator.

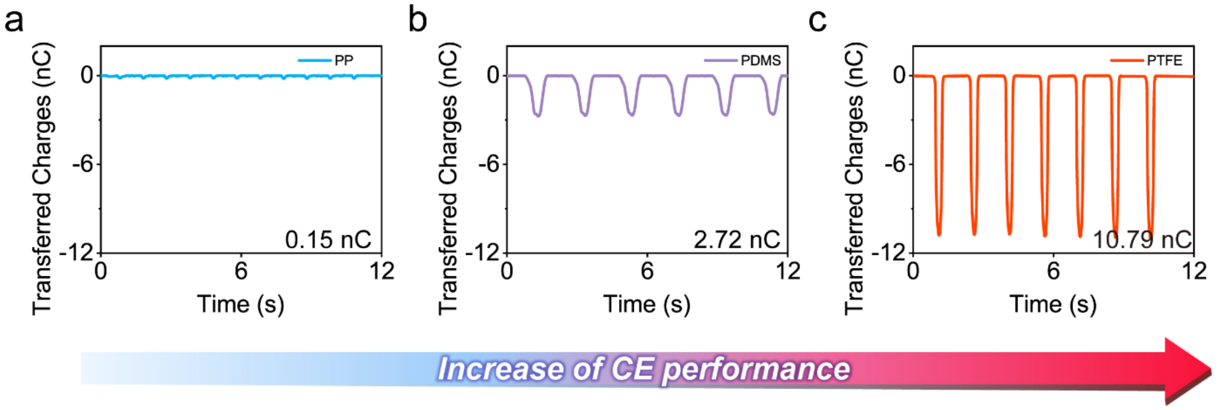

Supplementary Fig. 2 |Measured transferred charges of a pristine polymer-based SE-TENG that is repeatedly immersed in ultrapure water. a) PP-based, b) PDMS-based, c) PTFE-based. Source data are provided as a Source Data file.

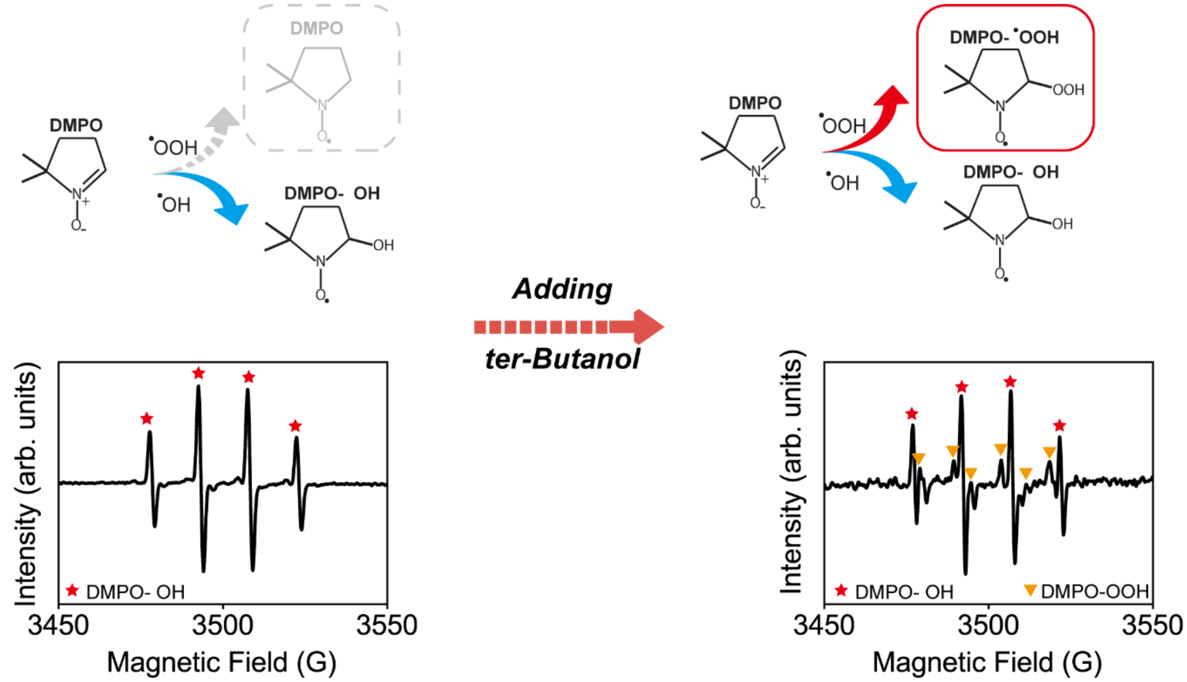

Supplementary Fig. 3 | Schematic illustration and corresponding measured profiles of the effect of introducing ter-butanol on the production of DMPO adducts. Source data are provided as a Source Data file.

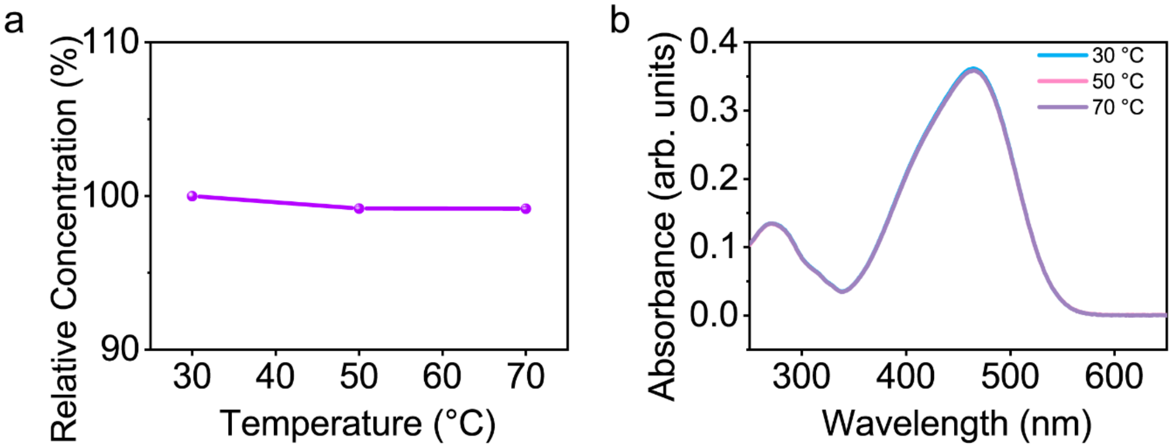

Supplementary Fig. 4 | Thermal stability test of methyl orange aqueous solution. a, Evolution of relative concentrations of MO aqueous solutions under various temperatures. **b,** Corresponding UV-Vis spectra. Error bars represent standard deviation based on three replicate data. Source data are provided as a Source Data file.

m/z	173	218	276	290	306	320
Degradation Products	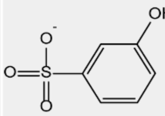	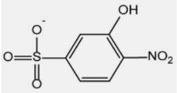	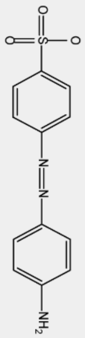	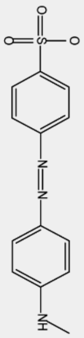	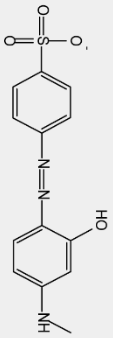	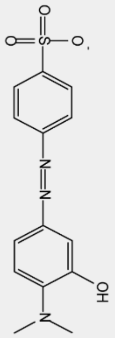

Supplementary Fig. 5 | The chemical structure of degradation products during the CEC degradation of methyl orange aqueous solution as identified by mass-spectroscopy.

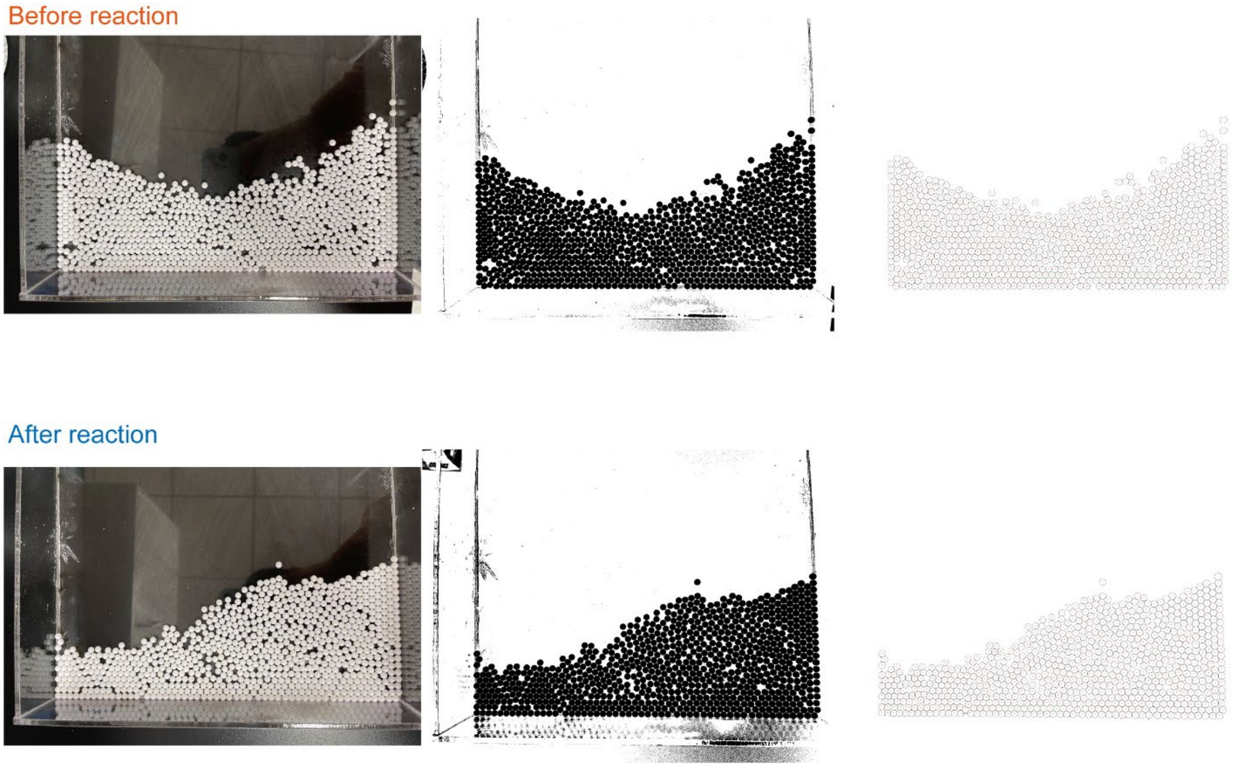

Supplementary Fig. 6 | Optical and corresponding Image J-processed images of PTFE milling balls before and after reactions.

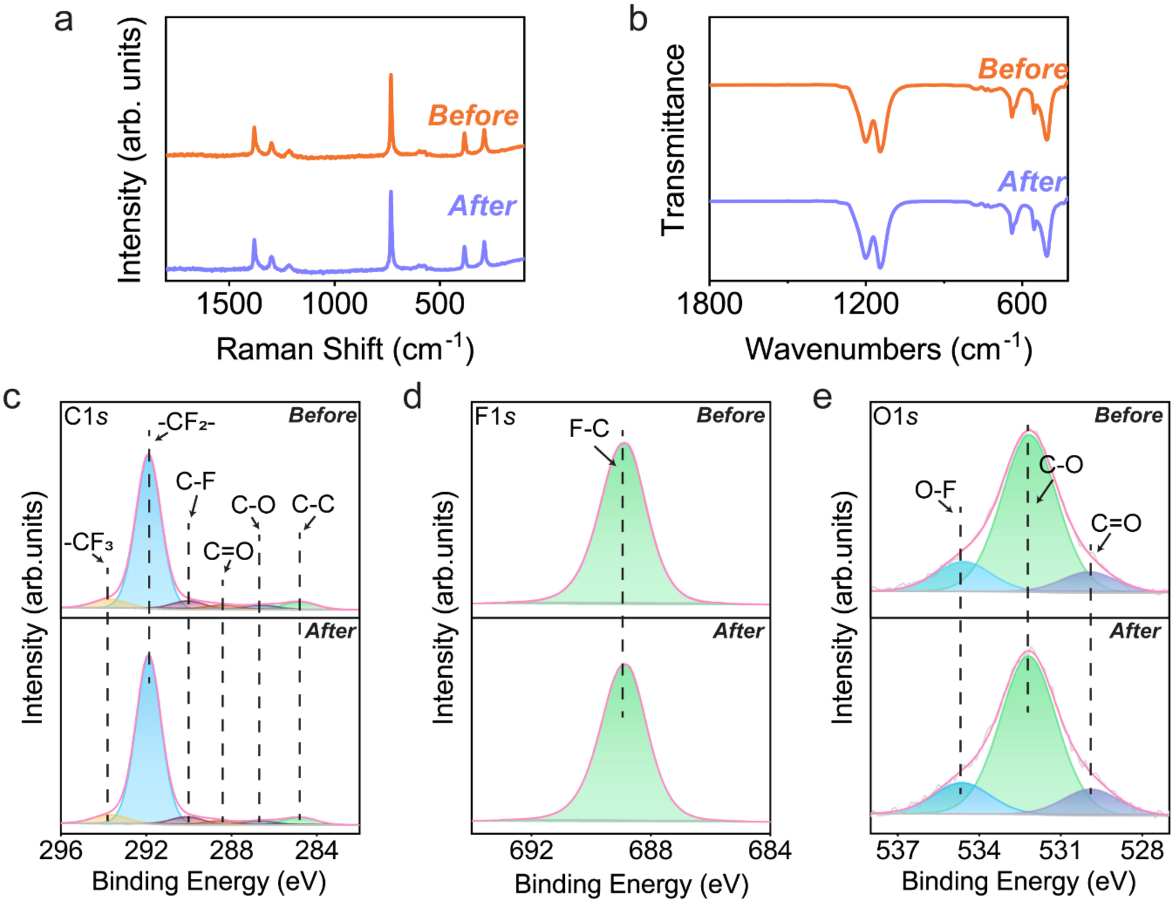

Supplementary Fig. 7 | Characterization of PTFE milling balls before and after contact-electro-catalysis. a, Raman spectra before (orange) and after (blue) the reaction. **b,** Fourier Transform Infrared (FTIR) spectra before (orange) and after (blue) the reaction. **c,** C1*s* **d,** F1*s* and **e,** O1*s* XPS spectra of PTFE balls before and after reaction. Source data are provided as a Source Data file.

a

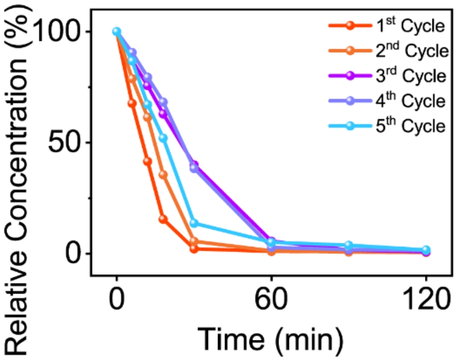

b

Cycle numbers	Degradation rate	Kinetic rate
1st cycle	99.35 %	0.0420
2nd cycle	99.45 %	0.0434
3rd cycle	99.12 %	0.0404
4th cycle	98.61 %	0.0356
5th cycle	98.25 %	0.0354

Supplementary Fig. 8 | Recyclability of PTFE milling balls during contact-electro-catalysis. **a,** Evolution of relative concentrations of MO aqueous solutions from 1 to 5 cycles. **b**, Summarized contact-electro-catalysis efficiency under different cycles. Source data are provided as a Source Data file.

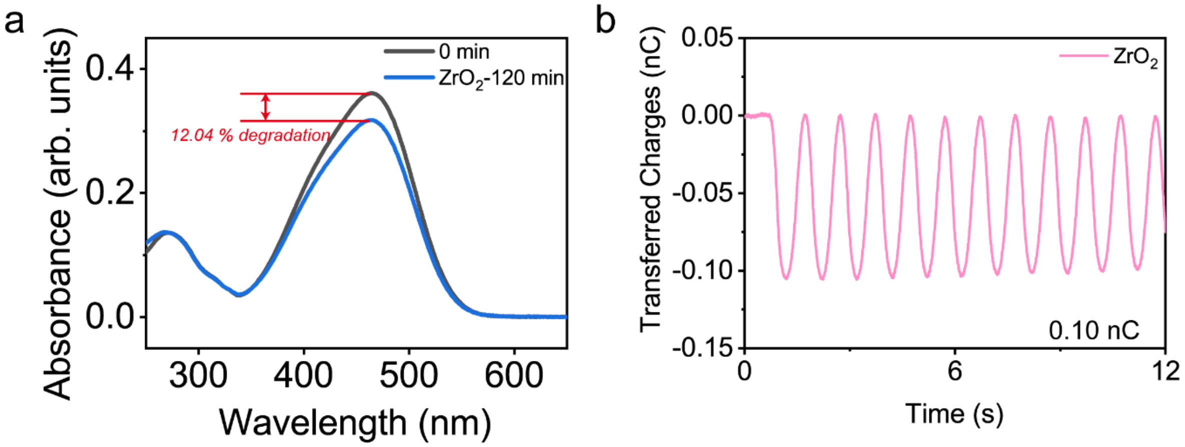

Supplementary Fig. 9 | Investigations on degradation by ZrO_2 and its CE ability. a, UV-Vis spectra of MO aqueous solution before and after grinding in ZrO_2 group for 120 mins. **b,** Measured transferred charges of a ZrO_2-based SE-TENG by repeatedly immersing it into ultrapure water. Source data are provided as a Source Data file.

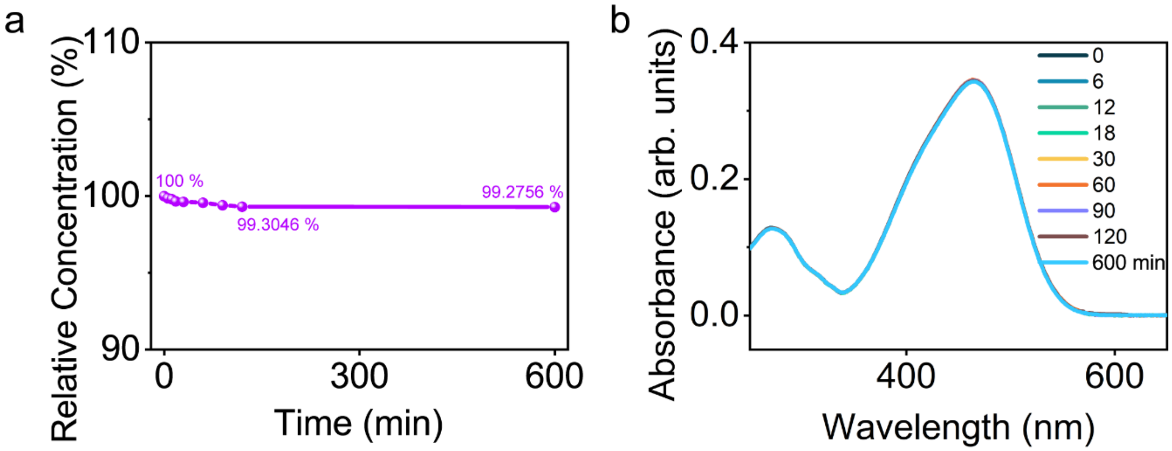

Supplementary Fig. 10 | Grinding at 50 RPM with prolonged milling time for methyl orange aqueous solution degradation. a, Evolution of relative concentration of MO aqueous solution under various milling intervals. **b,** Corresponding UV-Vis spectra. Source data are provided as a Source Data file.

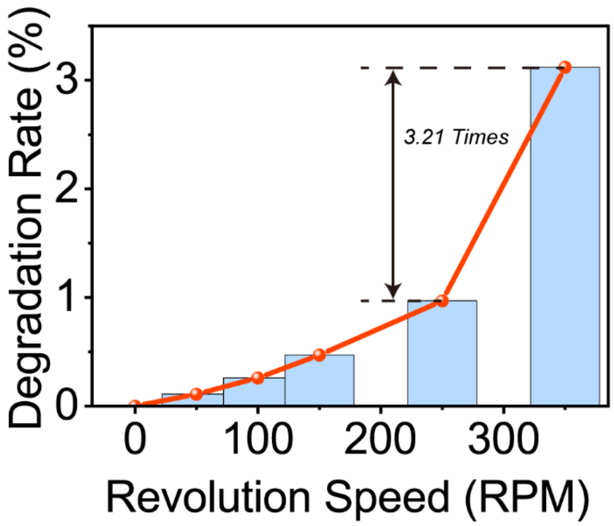

Supplementary Fig. 11 | Magnified illustration of degrading methyl orange aqueous solution in PP group under different revolution speeds. Source data are provided as a Source Data file.

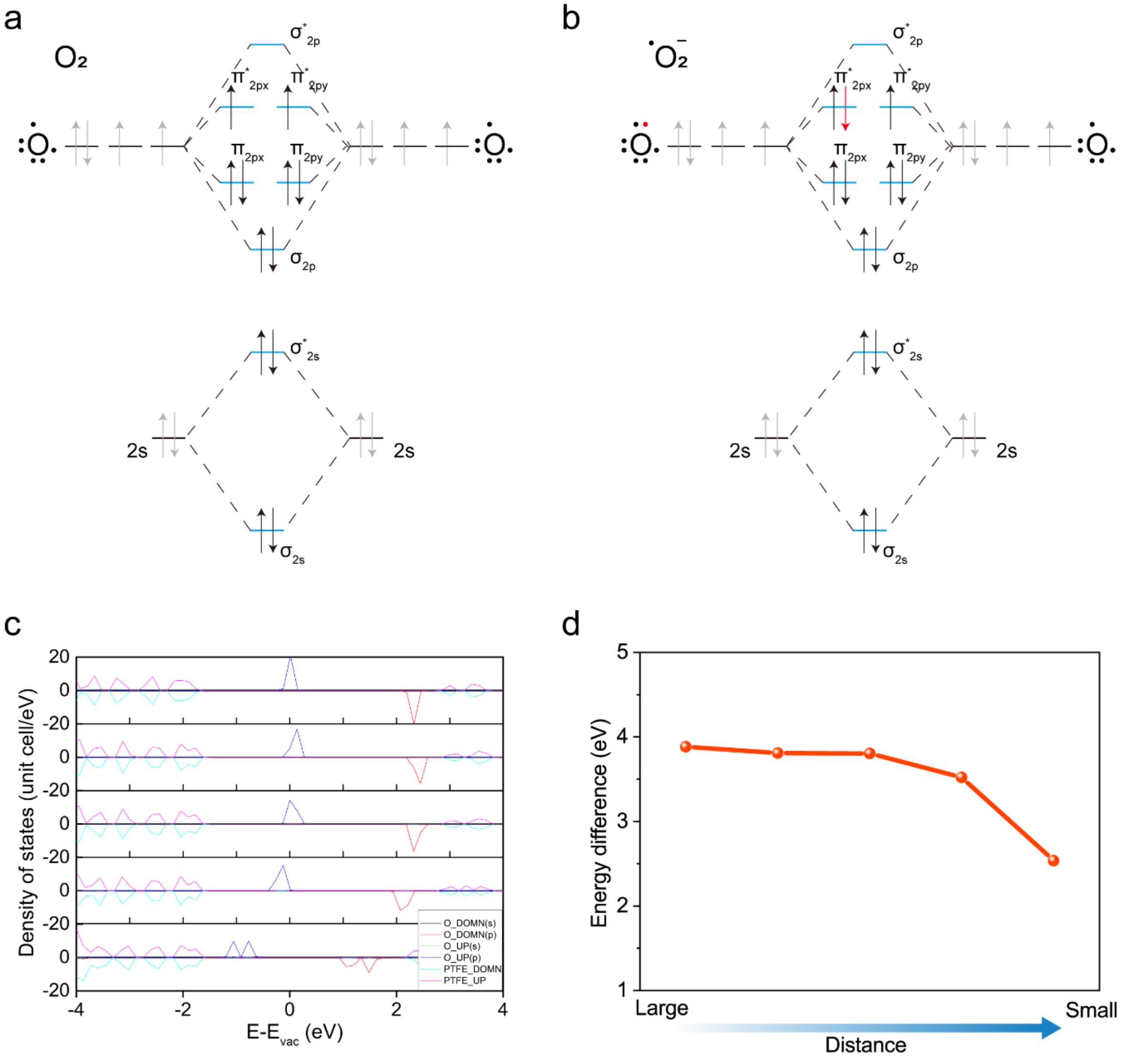

Supplementary Fig. 12 | Theoretical analysis of electron transfer process between PTFE and O_2. **a**, Molecular orbital configuration of oxygen molecules. **b**, Molecular orbital configuration of superoxide radicals. **c**, Partial density of state (PDOS) of oxygen and total density of state (TDOS) of PTFE in a co-existence model with O_2. **d**, Calculated energy difference between HOMO energy level of PTFE and antibonding π orbit of O_2 molecules at varying distances. Source data are provided as a Source Data file.

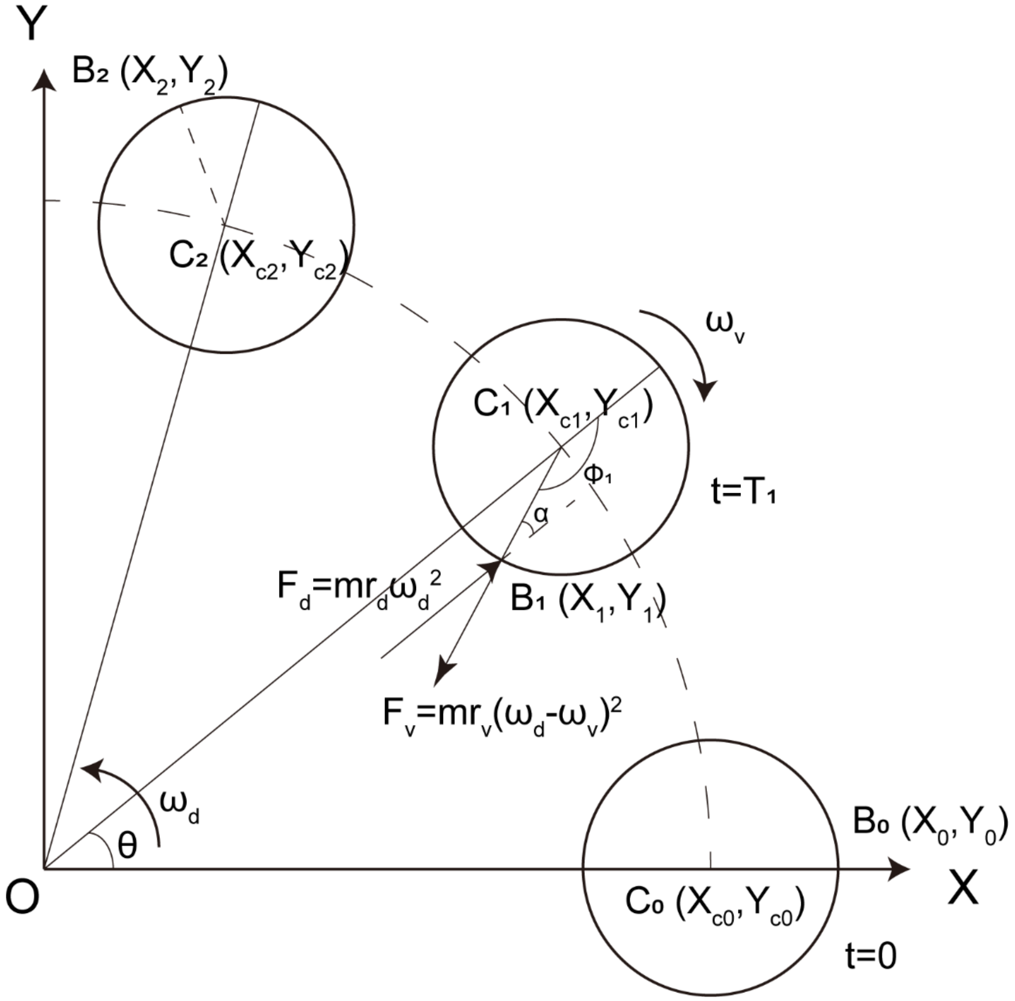

Supplementary Fig. 13 | Schematic illustration for the theoretical analysis of a planetary milling process.

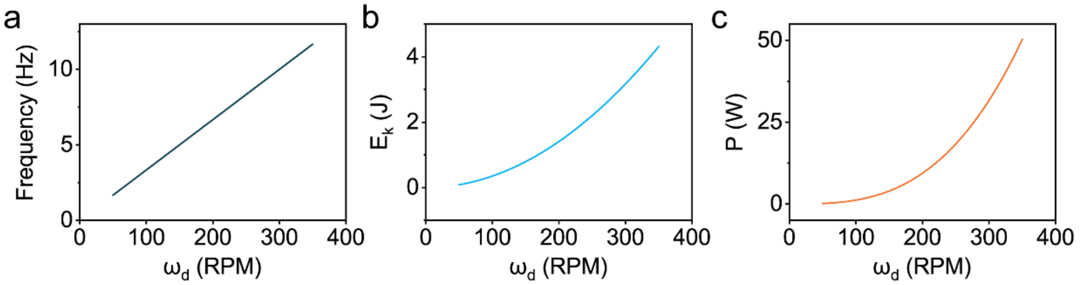

Supplementary Fig. 14 | Numerically calculated ball motion and impact under various revolution speeds. a, Frequency. **b,** Kinetic energy. **c,** Total power. Source data are provided as a Source Data file.

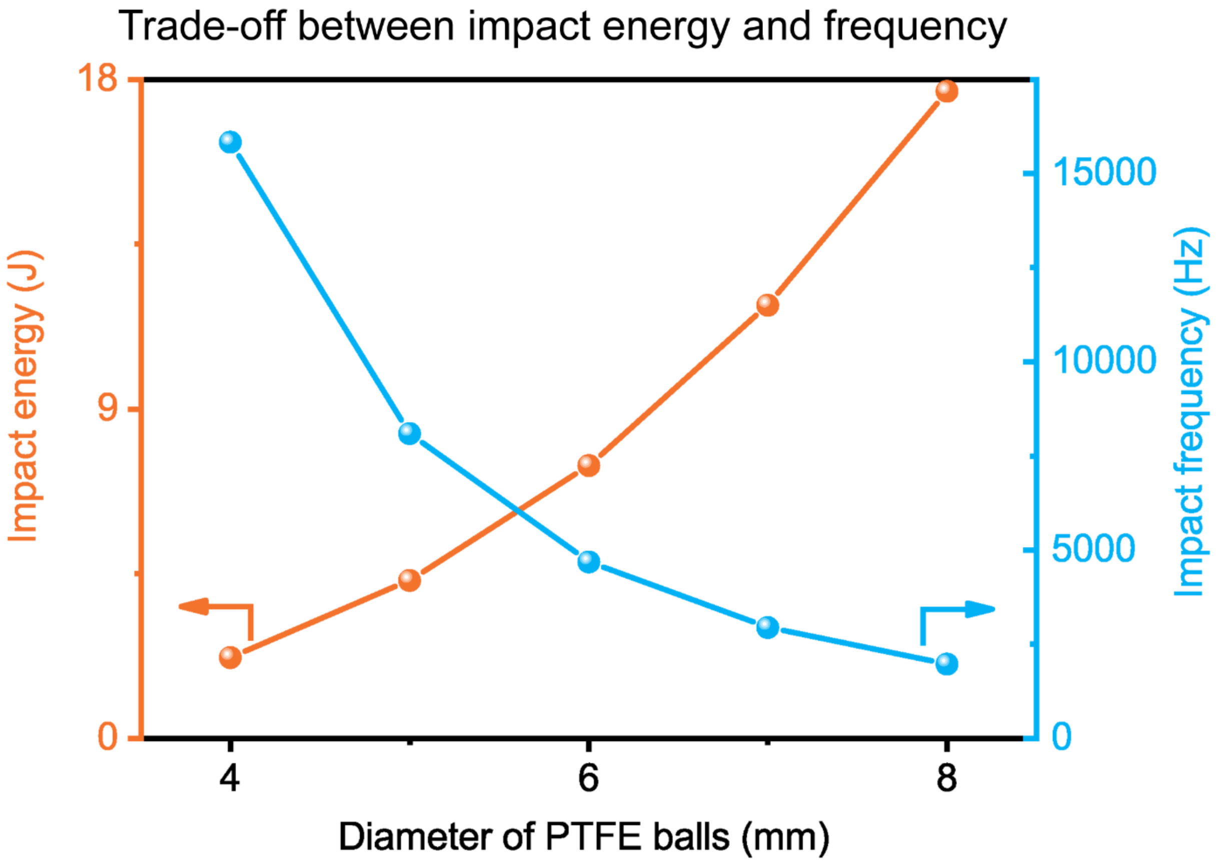

Supplementary Fig. 15 | Calculated evolution of impact energy and frequency when the diameter of PTFE balls varies from 4 to 8 mm. Note that the total weight of PTFE balls is given as 100 g. Source data are provided as a Source Data file.

Supplementary References

1. P. P. Chattopadhyay, I. Manna, S. Talapatra, S. K. Pabi, A mathematical analysis of milling mechanics in a planetary ball mill. *Mater. Chem. Phys.* **68**, 85-94 (2001).
2. J. P. Perdew, K. Burke, M. Ernzerhof, Generalized Gradient Approximation Made Simple. *Phys. Rev. Lett.* **77**, 3865-3868 (1996).
3. P. E. Blöchl, Projector augmented-wave method. *Phys. Rev. B* **50**, 17953-17979 (1994).
4. G. Kresse, D. Joubert, From ultrasoft pseudopotentials to the projector augmented-wave method. *Phys. Rev. B* **59**, 1758-1775 (1999).
5. X.-F. Li et al., Contaction of atoms for outstanding dielectric characteristics in KX-passivated polymer dielectrics. *Nano Energy* **107**, 108152 (2023).
6. X.-F. Li et al., Insights Into to the KX (X = Cl, Br, I) Adsorption-Assisted Stabilization of CsPbI2Br Surface. *Small* **18**, 2202623 (2022).
